# Chronic Proinflammatory Signaling Accelerates the Rate of Degeneration in a Spontaneous Polygenic Model of Inherited Retinal Dystrophy

**DOI:** 10.3389/fphar.2022.839424

**Published:** 2022-03-21

**Authors:** T. J. Hollingsworth, Xiangdi Wang, William A. White, Raven N. Simpson, Monica M. Jablonski

**Affiliations:** ^1^ Hamilton Eye Institute, Department of Ophthalmology, University of Tennessee Health Science Center, Memphis, TN, United States; ^2^ Department of Anatomy and Neurobiology, University of Tennessee Health Science Center, Memphis, TN, United States; ^3^ Department of Pharmaceutical Sciences, University of Tennessee Health Science Center, Memphis, TN, United States; ^4^ Department of Genetics, Genomics and Informatics, University of Tennessee Health Science Center, Memphis, TN, United States

**Keywords:** inherited retinal dystrophy, inflammation, microglia, TNFα, JAK/STAT, NF-κB, NLRP3

## Abstract

Collectively, retinal neurodegenerative diseases are comprised of numerous subtypes of disorders which result in loss of a varying cell types in the retina. These diseases can range from glaucoma, which results in retinal ganglion cell death, to age-related macular degeneration and retinitis pigmentosa, which result in cell death of the retinal pigment epithelium, photoreceptors, or both. Regardless of the disease, it’s been recently found that increased release of proinflammatory cytokines and proliferation of active microglia result in a remarkably proinflammatory microenvironment that assists in the pathogenesis of the disease; however, many of the details of these inflammatory events have yet to be elucidated. In an ongoing study, we have used systems genetics to identify possible models of spontaneous polygenic age-related macular degeneration by mining the BXD family of mice using single nucleotide polymorphism analyses of known genes associated with the human retinal disease. One BXD strain (BXD32) was removed from the study as the rate of degeneration observed in these animals was markedly increased with a resultant loss of most all photoreceptors by 6 months of age. Using functional and anatomical exams including optokinetic nystamography, funduscopy, fluorescein angiography, and optical coherence tomography, along with immunohistochemical analyses, we show that the BXD32 mouse strain exhibits a severe neurodegenerative phenotype accompanied by adverse effects on the retinal vasculature. We also expose the concurrent establishment of a chronic proinflammatory microenvironment including the TNFα secretion and activation of the NF-κB and JAK/STAT pathways with an associated increase in activated macrophages and phagoptosis. We conclude that the induced neuronal death and proinflammatory pathways work synergistically in the disease pathogenesis to enhance the rate of degeneration in this spontaneous polygenic model of inherited retinal dystrophy.

## Introduction

Progressive retinal dystrophies (RDs) result in the loss of the rod and cone photoreceptors, though, depending on the disease itself, the degeneration observed will occur at varying rates with specific cell types affected more so than others (Cremers et al., 2004; Hunt et al., 2004; Hollingsworth and Gross, 2012; Iannaccone et al., 2015; Rho et al., 2021). For example, diseases like age-related macular degeneration (AMD) and Stargardt’s disease primarily or initially affect cone photoreceptors, the retinal pigment epithelium (RPE), or both, with the rates and onsets of degeneration varying dramatically between the two (Cremers et al., 2004; Hunt et al., 2004; Al-Zamil and Yassin, 2017; Fleckenstein et al., 2021; Rho et al., 2021). Contrarily, retinitis pigmentosa (RP) and Leber congenital amaurosis (LCA) both affect rod photoreceptors first, and ultimately the cones as well, while advancing at rates varying from gene to gene and person to person (Rivolta et al., 2002; den Hollander et al., 2008; Ferrari et al., 2011; Hollingsworth and Gross, 2012; O'Neal and Luther, 2021). Although RDs are incredibly heterogenous, some common pathogenetic features have been observed to be conserved amongst them. Regardless of the initial etiology of an RD, proinflammatory signaling has been observed in the retinas of RD models including those for AMD, RP, LCA, and even glaucoma (Brown and Neher, 2012; Soto and Howell, 2014; Iannaccone et al., 2015; Appelbaum et al., 2017; Iannaccone et al., 2017; Silverman and Wong, 2018; Wooff et al., 2019; Hollingsworth and Gross, 2020; Ucgun et al., 2020; Fleckenstein et al., 2021; Hollingsworth et al., 2021). Previously, we have shown upregulation of multiple proinflammatory and autoinflammatory pathways in multiple models of rhodopsin-mediated RP (Hollingsworth and Gross, 2020; Hollingsworth et al., 2021). While these models do recapitulate RP phenotypes, they are hardly representative of a truly natural model of the disease; those mice bear full human rhodopsin genes harboring one single point mutation that was introduced via knock-in methods (Hollingsworth and Gross, 2013; Sandoval et al., 2014). During a study using systems genetics and the BXD family of mice to attempt to elucidate spontaneous high-fidelity models of AMD, we discovered a novel, spontaneous model of a fairly rapid RD, BXD32, which is likely polygenic due to the inherent nature of the BXD family of mice (Geisert and Williams, 2020; Ashbrook et al., 2021). BXD mice are a family of recombinant inbred strains that were derived by crossing a female C57BL/6J and male DBA/2J, two very common mouse strains, and consecutively inbreeding the F2 progeny for more than 20 generations (Geisert and Williams, 2020; Ashbrook et al., 2021). This allowed for homologous recombination to occur at will, making each fully inbred strain genetically distinct. The DBA/2J genome has more than 5 million SNPs, greater than 400,000 insertions/deletions and multiple copy number variants compared to C57BL/6J and these all differentially segregate in each BXD strain due to the natural homologous recombination. Fortunately, each BXD strain has had its genome fully sequenced. It was the purpose of this study to both discern the pathological degenerative phenotypes in the BXD32 mouse strain and determine if chronic proinflammatory activation occurs in the retinas of these mice, allowing for future design of possible therapeutics to intervene in the pathogenesis observed in these mice.

## Materials and Methods

### Animals

C57BL/6J and BXD32 mice were obtained from the Jackson Laboratories. All animals were used in accordance with the Association for Research in Vision and Ophthalmology (ARVO) and University of Tennessee Health Science Center Institutional Animal Care and Use Committee.

### Optokinetic Nystagmography

WT and BXD32 mice (n = 3-4 mice/strain) at p63, p84, p105, p126, p147, and p168 were placed onto the pedestal in an OptoDrum OKN machine (Stoelting, 620 Wheat Lane, Wood Dale, IL, United States). The visual acuity (VA) data was collected by fixing the rotation speed on 12°/s and fixing the contrast on 99.72%. The contrast sensitivity (CS) result was collected by fixing the rotation speed on 12°/s and fixing the cycles on 0.103 cycles/degree. Statistical analysis performed using two-tailed student *t* test.

### Optical Coherence Tomography

WT and BXD32 mice (n = 3-4mice/strain) at p63, p84, p105, p126, p147, and p168 were anesthetized using ketamine/xylazine (intraperitoneally 71.42 mg/kg ketamine/14.3 mg/kg xylazine in PBS, pH 7.4) and pupils dilated using 1% tropicamide. To keep the eyes lubricated and maintain corneal clarity, artificial tears (Systane Ultra) were applied when needed. The mice were subsequently examined by OCT using an Eyemera OCT (IIScience, 3003 N 1st., San Jose, CA, United States) through the optic nerve head with the purpose of visualizing total retinal and ONL thicknesses non-invasively. Quantification of ONL thickness performed from histological sections through the optic nerve head (ONH).

### Funduscopy/Fluorescein Angiography

WT and BXD32 (n = 3-4mice/strain) mice at p63, p84, p105, p126, p147, and p168 were anesthetized using ketamine/xylazine (intraperitoneally 71.42 mg/kg ketamine/14.3 mg/kg xylazine in PBS, pH7.4) and eyes dilated using 1% tropicamide. The animals were then intraperitoneally injected with 100 μl of 4% fluorescein and subsequently imaged with either white light (funduscopy for examining retinal pallor/pigmentation) or 488 nm light (fluorescein angiography for vascular anomalies) emitted from an Eyemera Fundus Camera (IIScience, 3003 N 1st., San Jose, CA, United States).

### Fluorescent Immunohistochemistry

Whole eyes from WT and BXD32 (n = 3-4mice/strain, 1 slide/mouse, 3 sections per slide) mice at p63, p84, p105, p126, p147, and p168 were enucleated and fixed in 4% paraformaldehyde in PBS, pH 7.4 overnight at 4°C. Fixation was quenched in 100 mM glycine in PBS, pH 7.4 for 10 min at room temperature and subsequently washed in PBS. Eyes were dehydrated with 30 min incubations in a graded ethanol series (50, 70, 85, 95 and 100%) then cleared via a 30 min incubation in graded xylenes (2:1, 1:1, and 1:2 ethanol: xylenes), and two 30 min incubations in 100% xylenes. Eyes were then infiltrated with paraffin using a graded paraffin series with 30 min incubations in 2:1, 1:1, and 1:2 xylenes:paraffin and two subsequent 1 h incubations in 100% paraffin. Paraffin-embedded tissue was then sectioned at 8 µm or 16 μm and sections deparaffinized and rehydrated, treated using heat-mediated antigen retrieval by heating slides at 95°C in sodium citrate buffer (10 mM sodium citrate, 0.05% Tween-20, pH 6.0) for 1 h, washed in PBS twice and subsequently blocked in 10% goat serum/5% BSA/0.5% TritonX-100 in PBS for 30 min at RT. Primary antibodies against markers for glial cells and proinflammatory signaling pathways were then applied at recommended dilutions and incubated overnight at 4°C (Table 1). Slides were then washed in PBS, pH 7.4 three times for 10 min each. Post-washing, slides were incubated in secondary antibodies conjugated to either AlexaFluor488, AlexaFluor568, AlexaFluor647, or horseradish peroxidase (A21121, A21241, A11036, A21236, A11006, A21245, A16078, G21234; ThermoFisher; 168 Third Avenue, Waltham, MA, United States) at 1:400 dilutions for 1 h and nuclei stained using a 1:10,000 dilution of 14.3 mM DAPI (D21490; ThermoFisher; 168 Third Avenue, Waltham, MA, United States). For phosphorylated STAT3 (pSTAT3) and STAT3, tyramide signal amplification (TSA) was performed using the TSA with SuperBoost kit with tyramide reagent conjugated to either AlexaFluor 488 or AlexaFluor568 (B40926, B40953, B40956; ThermoFisher; 168 Third Avenue, Waltham, MA, United States) following the manufacturer’s instructions. Slides were then washed in PBS, pH 7.4 four times and mounted using Prolong Diamond Antifade mountant (P36961; ThermoFisher; 168 Third Avenue, Waltham, MA, United States). For TUNEL labeling, the Click-It Plus TUNEL Kit (C10647; ThermoFisher; 168 Third Avenue, Waltham, MA, United States) was used to label apoptotic nuclei with AlexFluor488 following the manufacturer’s instructions. After drying overnight, sections were imaged using a Zeiss 710 laser scanning confocal microscope (LSM) using a 40X objective with 1.3 numerical aperture (NA) or 63X objective with 1.4 NA with an associated 1.6X zoom (100X). Fluorescent intensities analyzed using ImageJ. Statistical analysis performed using two-tailed Welch’s *t* test (unequal variance).

**TABLE 1:** Antibodies used for IHC labeling of retinal sections.

Antibody Target	Catalog #	Host/IgG Isoform	Dilution	Source
GFAP	3670	Mouse IgG_1_	1:250	Cell Signaling Tech
GS	610518	Mouse IgG_2a_	1:500	BD Biosciences
IBA1	17198	Rabbit IgG	1:250	Cell Signaling Tech
RHO	MAB5356	Mouse IgG_1_	1:500	EMD Millipore
NF-κB p65	8242	Rabbit IgG	1:400	Cell Signaling Tech
NLRP3	MA5-23919	Rat IgG_2a_	1:250	ThermoFisher
TNFα	60291-1-Ig	Mouse IgG_2b_	1:250	ProteinTech
STAT3	9139	Mouse IgG_2a_	1:200	Cell Signaling Tech
pSTAT3-Tyr705	9145	Rabbit IgG	1:200	Cell Signaling Tech
pSTAT3-Ser727	9134	Rabbit IgG	1:200	Cell Signaling Tech
SOCS3	ab16030	Rabbit IgG	1:100	abcam

## Results

### OKN Reveals a Loss of Visual Acuity and Contrast Sensitivity in the BXD32 Mouse

**FIGURE 1:**
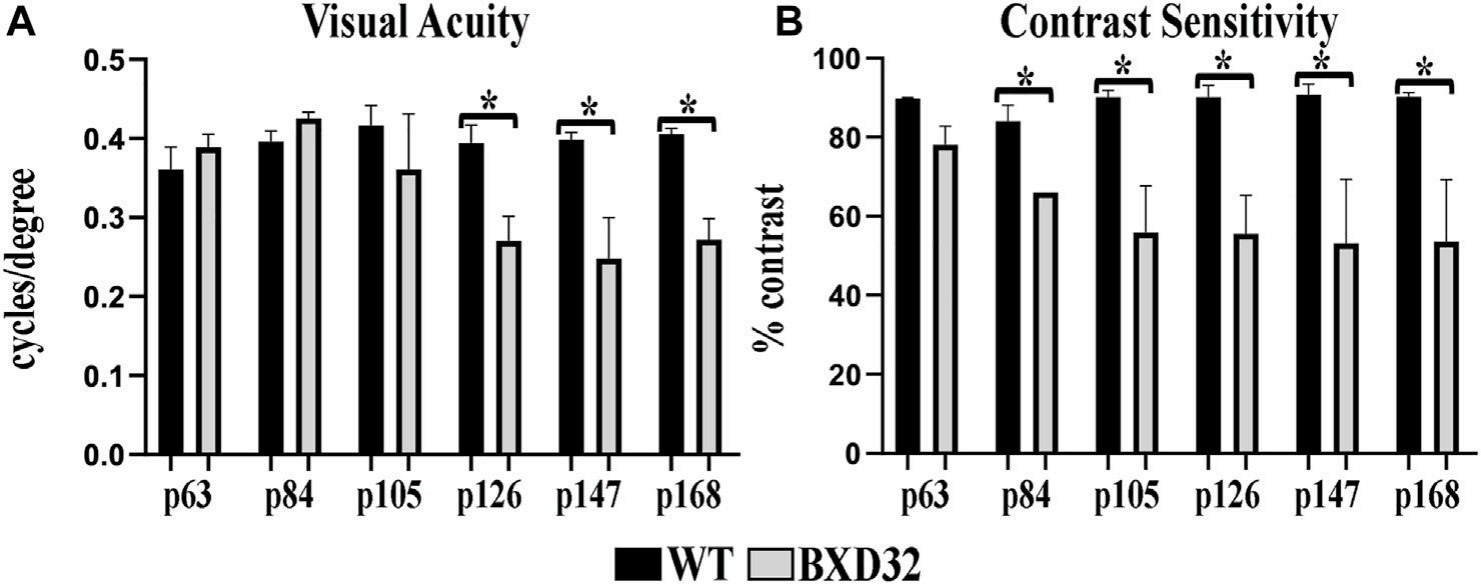
The BXD32 mouse retina exhibits diminished visual ability by OKN. WT and BXD32 mice were examined using OKN to assess for visual acuity **(**VA, **A)** and contrast sensitivity **(**CS**, B)**, respectively. BXD32 mice exhibit a loss of visual acuity beginning around p105 and dropping significantly by p168. Similarly, but beginning much earlier at p63, the BXD32 mice lose contrast sensitivity compared to WT mice to levels roughly half of WT animals by p168. Error bars = ±SEM. *, *p* < 0.05.

Using screens with passing alternating black and white bars of varying thicknesses and at varying speeds, OKN data output allows for quantification of both the visual acuity (VA) and contrast sensitivity (CS) of the subject, both of which decrease with age normally. BXD32 mice, while initially having similar VA (Figure 1A) and CS (Figure 1B) as WT, gradually lose both with age more rapidly than observed in WT, correlating to a loss of photoreceptors in the outer retina.

### OCT Analysis Reveals a Rapid Loss of Photoreceptor Cells in the BXD32 Mouse Retina

**FIGURE 2:**
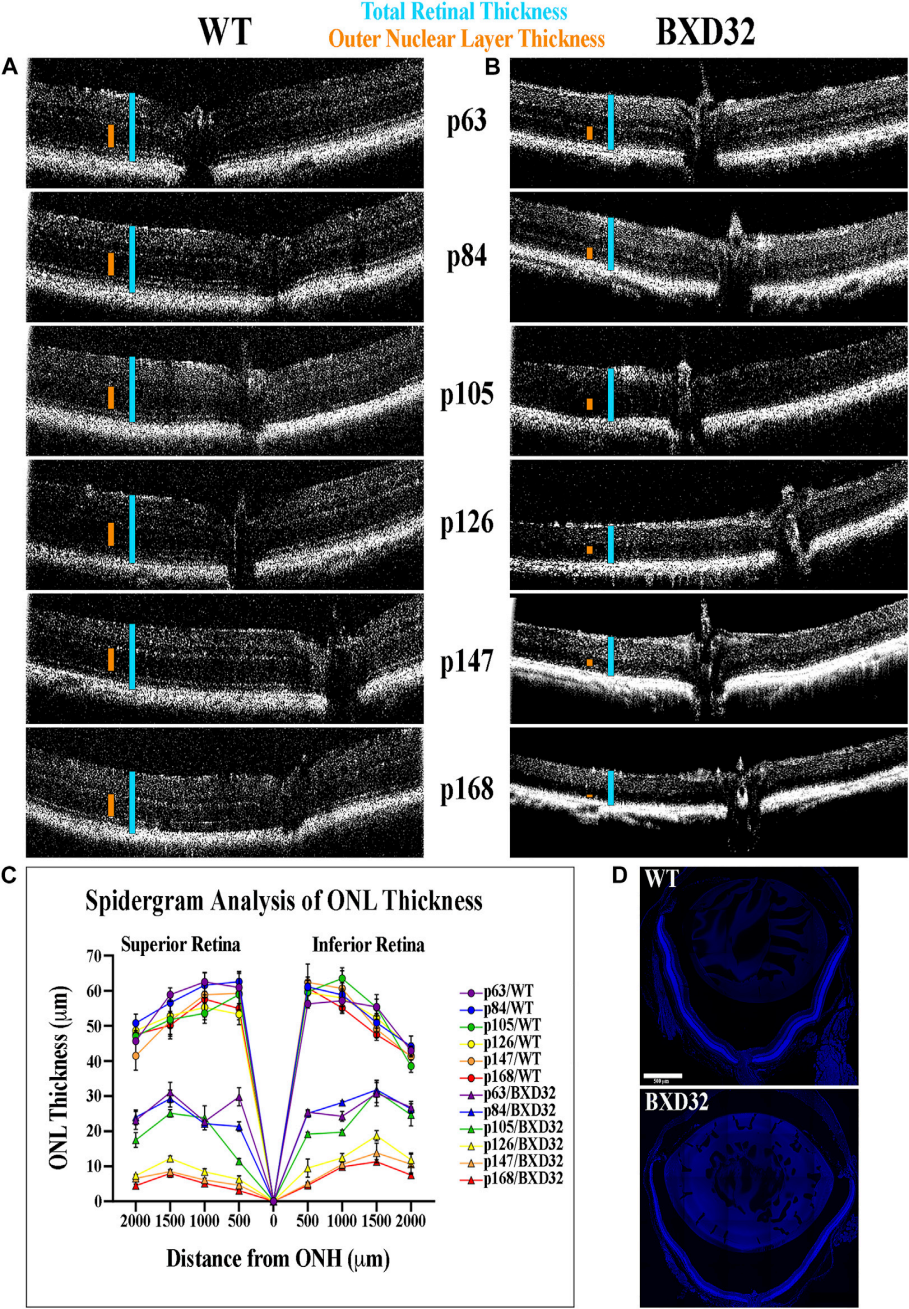
BXD32 retinas degenerate rapidly when observed by OCT. WT **(A)** and BXD32 **(B)** mice were anesthetized and had their retinas imaged by OCT. p63 BXD32 mice already demonstrate thinning of both the ONL (orange caliper) and the total retina (light blue caliper) compared to WT. This thinning increases rapidly until almost no ONL exists by p168. **(C)** ONL measurements were taken from tiled DAPI stained retinal sections through the ONH at distances of 500, 1,000, 1,500, and 2000 μm superiorly and inferiorly to the ONH and graphed as a spidergram. Examples of typical retinal tiled images are shown in **(D)**.

By utilizing infrared light passing through the cornea, OCT can generate an image of the retinal tissue through interpolation of the photons reflected back to the lens by the tissue, the amount of which is determined by the density of the retinal layers. Using OCT imaging (Figures 2A,B), we are able to visualize a rapid, early onset loss of the outer layers of the retina, namely the photoreceptor outer nuclear layer (ONL) and inner and outer segments, resulting in a loss of total retinal thickness. This finding strongly correlates to a severe form of inherited retinal dystrophy (IRD). Using histological sections through the ONH, ONL measurements show the retinal degeneration in the BXD32 mouse occurs superiorly to inferiorly and centrally to peripherally with the peak rate of degeneration occurring around p105 (Figures 2C,D).

### Funduscopy and Fluorescein Angiography Reveal Hallmarks of Severe IRD

**FIGURE 3:**
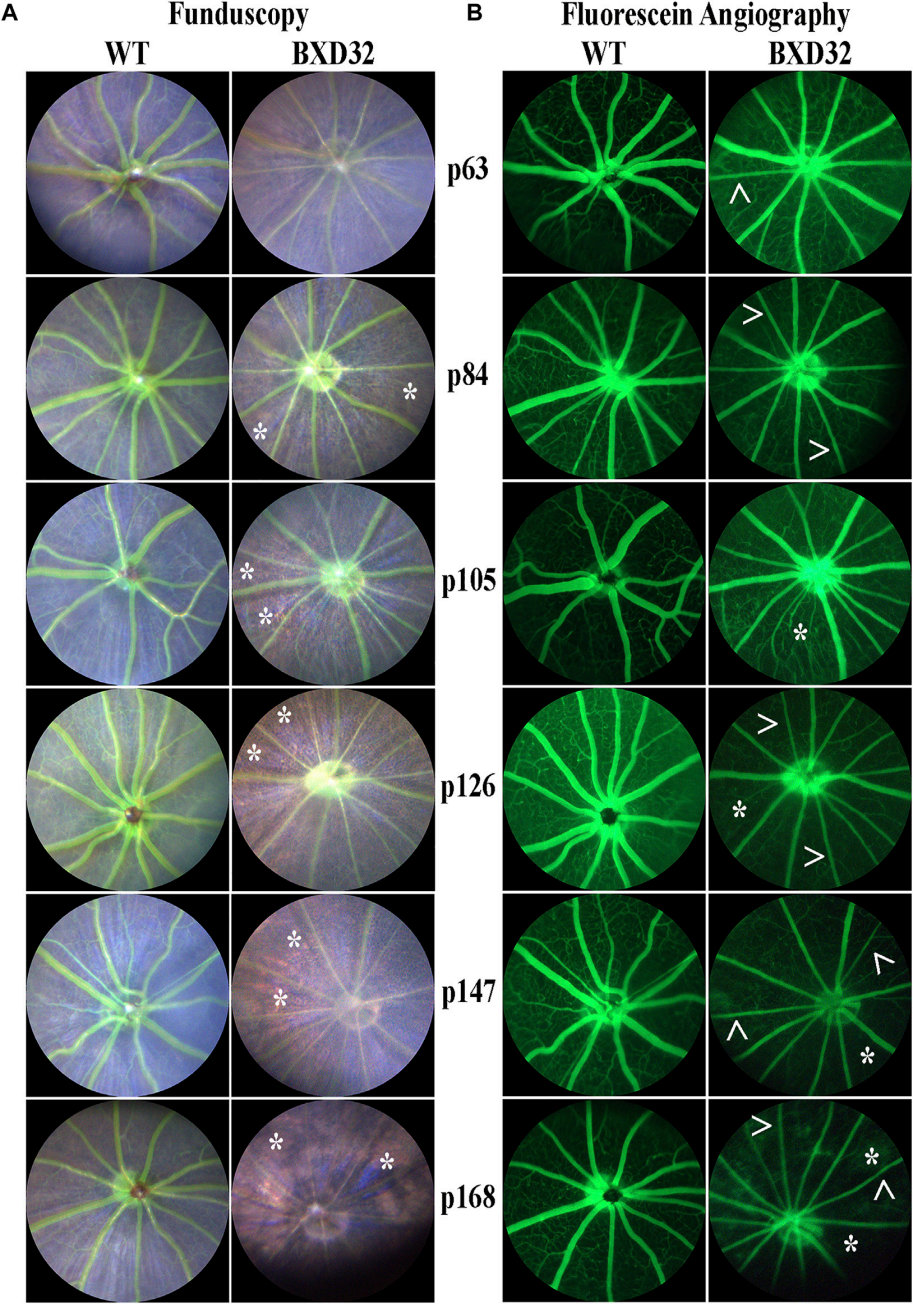
Funduscopy and fluorescein angiography reveal retinal phenotypes consistent with RP. WT and BXD32 retinas were observed under white light for funduscopy **(A)** and using 488 nm for fluorescein angiography **(B)** light post-fluorescein injection. By funduscopy, BXD32 mice as early as p84 begin showing RPE through the neural retina (asterisks, *) indicating a major loss of photoreceptors in these regions. Fluorescein angiography reveals vessel attenuation in the superficial vascular plexus (arrowheads, >) beginning around p63 with some regions exhibiting vaso-obliteration, mostly in the intermediate and deep vascular plexi (asterisk, *).

To observe the overall health of the retina visually, funduscopy is used by shining a white light into the eye, allowing for full retinal visualization. By injecting a subject with fluorescein and visualizing the retina with a blue (∼488 nm) light, one is also capable of examining the retinal vasculature. The BXD32 mouse retina shows strong dissimilarity to WT retinas, which will tend toward a healthy bluish pallor. The BXD32 retina, instead, exhibits distinct features of RP with a loss of retinal thickness allowing for the RPE to be visualized (Figure 3A). Observation of the retinal vasculature reveals vessel attenuation with age, indicative of an RP phenotype as well (Figure 3B). No vascular leakage was witnessed at any age; however, an apparent loss of visible intermediate and deep vascular plexi can be observed with age.

### BXD32 Mouse Retinas Exhibit Markedly Up-Regulated Levels of Both Monocytic Activation and Invasion and Proinflammatory Pathway Proteins

**FIGURE 4:**
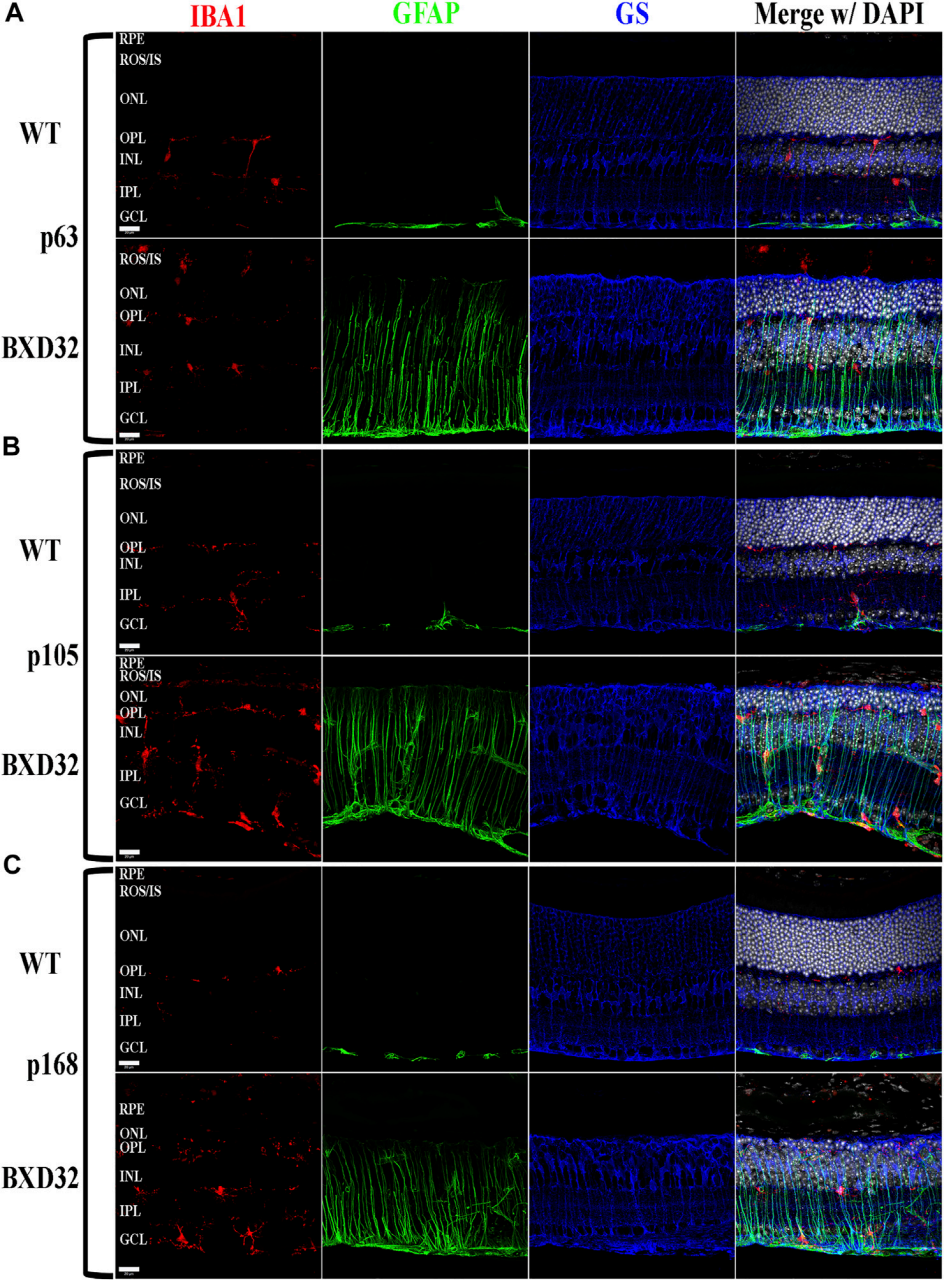
BXD32 mouse retinas have advanced glial hypertrophy and an increased presence of monocytes in the outer retina. WT and BXD32 retinal sections were immunolabeled for glial cells using IBA1 (red), GFAP (green), and GS (blue). Beginning at the earliest timepoint examined p63 **(A)**, BXD32 retinas exhibit dramatic upregulation of GFAP and a larger number of monocytes, presumably microglia, both in the outer retina and the retina as a whole. By p105 **(B)**, no decrease in GFAP is observed and monocyte numbers are even higher than at p63. At p168 **(C)**, the outer retina is mostly degenerated and while monocyte numbers are lesser from p105, GFAP is still upregulated, indicating continued retinal stress. GS levels remained relatively similar amongst WT mice and BXD32 mice, with occasional variations with age. Nuclei stained with DAPI (white/grey). RPE, retinal pigment epithelium; ROS/IS, rod outer segments/inner segments; ONL, outer nuclear layer; OPL, outer plexiform layer; INL, inner nuclear layer; IPL, inner plexiform layer; GCL, ganglion cell layer. Scale bars = 20 μm.

**FIGURE 5:**
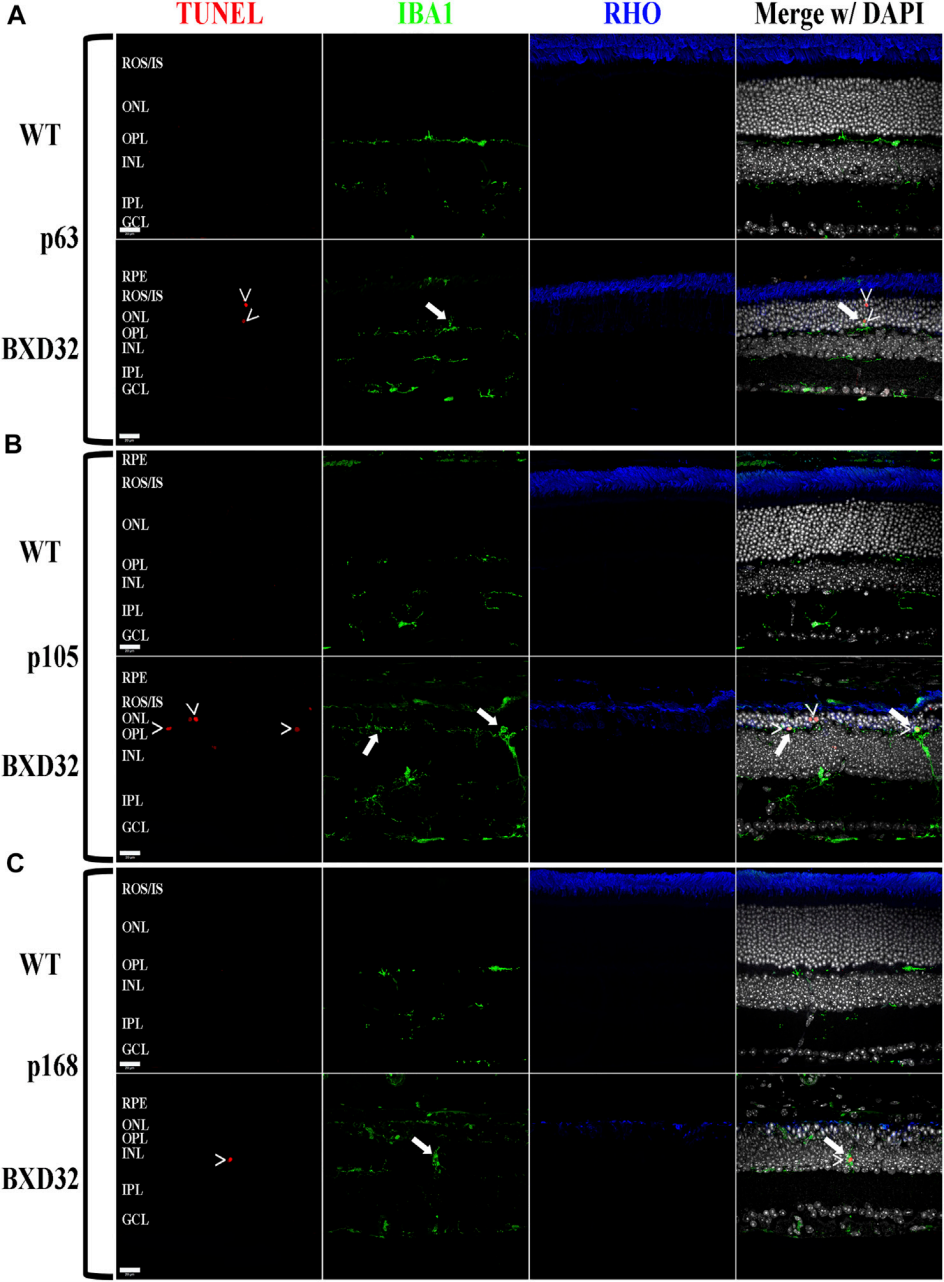
The BXD32 retina exhibits cell death by apoptosis and proper function of microglia. Using TUNEL labeling (red) for apoptotic nuclei (arrowheads, **>**) and immunolabeling for monocytes (IBA1, green) and photoreceptors (RHO, blue) at p63 **(A)**, p105 **(B)** and p168 **(C)**, presumed microglia can be observed migrating to the ONL and phagocytosing apoptotic photoreceptor nuclei (arrows) the normal function of microglial cells in the retina under degenerative conditions. Nuclei stained with DAPI (white/grey). RPE, retinal pigment epithelium; ROS/IS, rod outer segments/inner segments; ONL, outer nuclear layer; OPL, outer plexiform layer; INL, inner nuclear layer; IPL, inner plexiform layer; GCL, ganglion cell layer. Scale bars = 20 μm.

**FIGURE 6:**
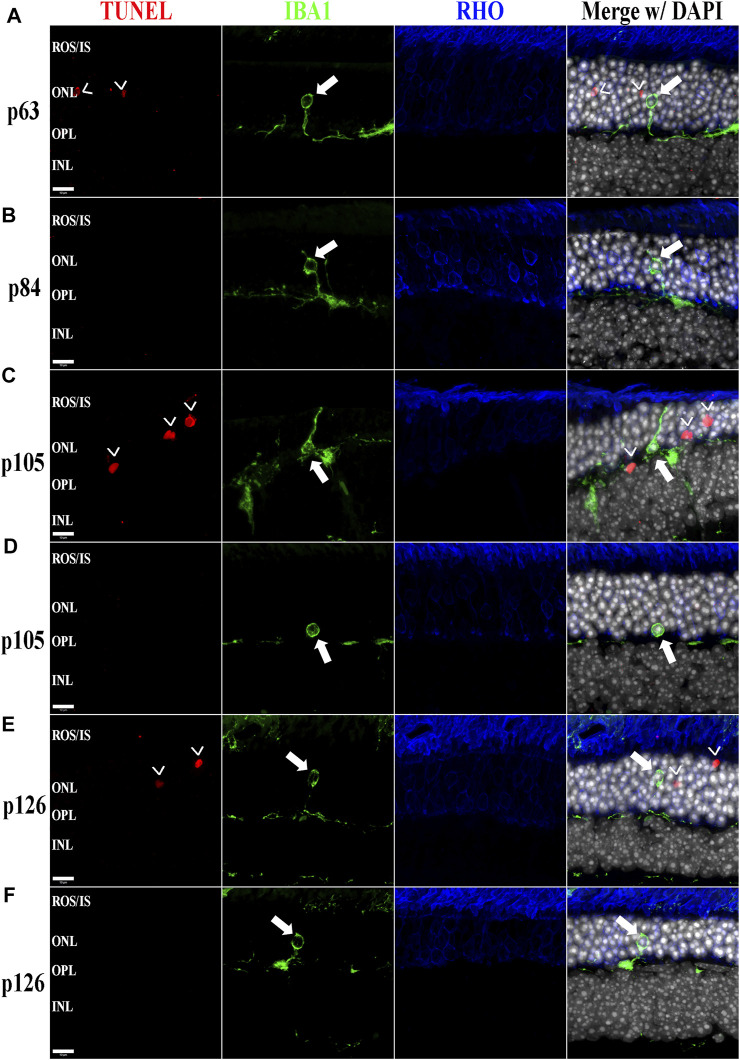
The BXD32 mouse retina shows aberrant phagocytosis of living photoreceptors by monocytes. Using TUNEL labeling (red) for apoptotic nuclei (arrowheads, **>**) and immunolabeling for monocytes (IBA1, green) and photoreceptors (RHO, blue) at p63 **(A)**, p84 **(B)**, p105 **(C,D)**, **and** p126 **(E,F)**, monocytes of either microglial or bloodborne origins can be observed phagocytosing non-apoptotic photoreceptors (arrows), a process known as phagoptosis. Nuclei stained with DAPI (white/grey). ROS/IS, rod outer segments/inner segments; ONL, outer nuclear layer; OPL, outer plexiform layer; INL, inner nuclear layer. Scale bars = 10 μm.

Previously, we and other labs have shown that as RDs progress, proinflammatory pathways are upregulated due to release of various cytokines including TNFα, IL-6, IL-1β, and others (Ten Berge et al., 2019; Wooff et al., 2019; Yi et al., 2019; Hollingsworth et al., 2021). To investigate whether the BXD32 mouse retina propagates the formation of a proinflammatory microenvironment, we paraffin embedded eyes, sectioned, and immunolabeled them for retinal stress markers, multiple proinflammatory cytokines/pathway proteins. Under normal physiological conditions, glial fibrillary acidic protein (GFAP) is expressed primarily in the nerve fiber layer regions of the retina and is localized to astrocytes. Under disease conditions, however, GFAP expression is increased and in turn localized to the Müller glial cells of the retina (Lewis and Fisher, 2003). This upregulation has been linked to both hypertrophy and gliosis in the retina. To test for GFAP upregulation, we performed fIHC on retinal sections from BXD32 and WT mice (Figure 4 and Supplementary Figure S1). GFAP upregulation starts from the earliest time point tested in the BXD32 mice and levels never diminish, indicating a highly stressed, detrimental retinal state. In addition to GFAP, we immunolabeled for glutamine synthetase (GS), a marker for Müller glia (as well as other glial cells), and IBA1, a marker for macrophages, or in the case of the central nervous system, microglia, the resident macrophages in the retina and brain. Normally, microglia are found resting in the inner retinal layers and present with a ramified morphology of filamentous projections that extend to the outer retina seeking out dying cells or foreign invaders (Li et al., 2015; Silverman and Wong, 2018; Rashid et al., 2019; Kinuthia et al., 2020). We observed a dramatic increase in the number of macrophages not only present in the retina, but migrating to the outer retina and RPE, something these cells only tend to do under disease conditions (Li et al., 2015; Rashid et al., 2019). In addition to labeling for these glial and microglial cells, we performed fIHC for rhodopsin in conjunction with IBA1 and TUNEL labeling to indicate apoptosing cells (Figure 5 and Supplementary Figure S2). TUNEL labeling increased with age, peaking at p105 and decreasing up to p168 where significantly fewer photoreceptors are observed (Supplementary Figure S3). We visualized both phagocytosis of TUNEL-labeled cells as well as the aberrant phagocytosis of living cells, known as phagoptosis (Figure 6 and Supplementary Figure S4 **)**, recently discovered to be an underlying cause of cell death in RD (Brown and Neher, 2014; Zhao et al., 2015; Hollingsworth et al., 2021).

**FIGURE 7:**
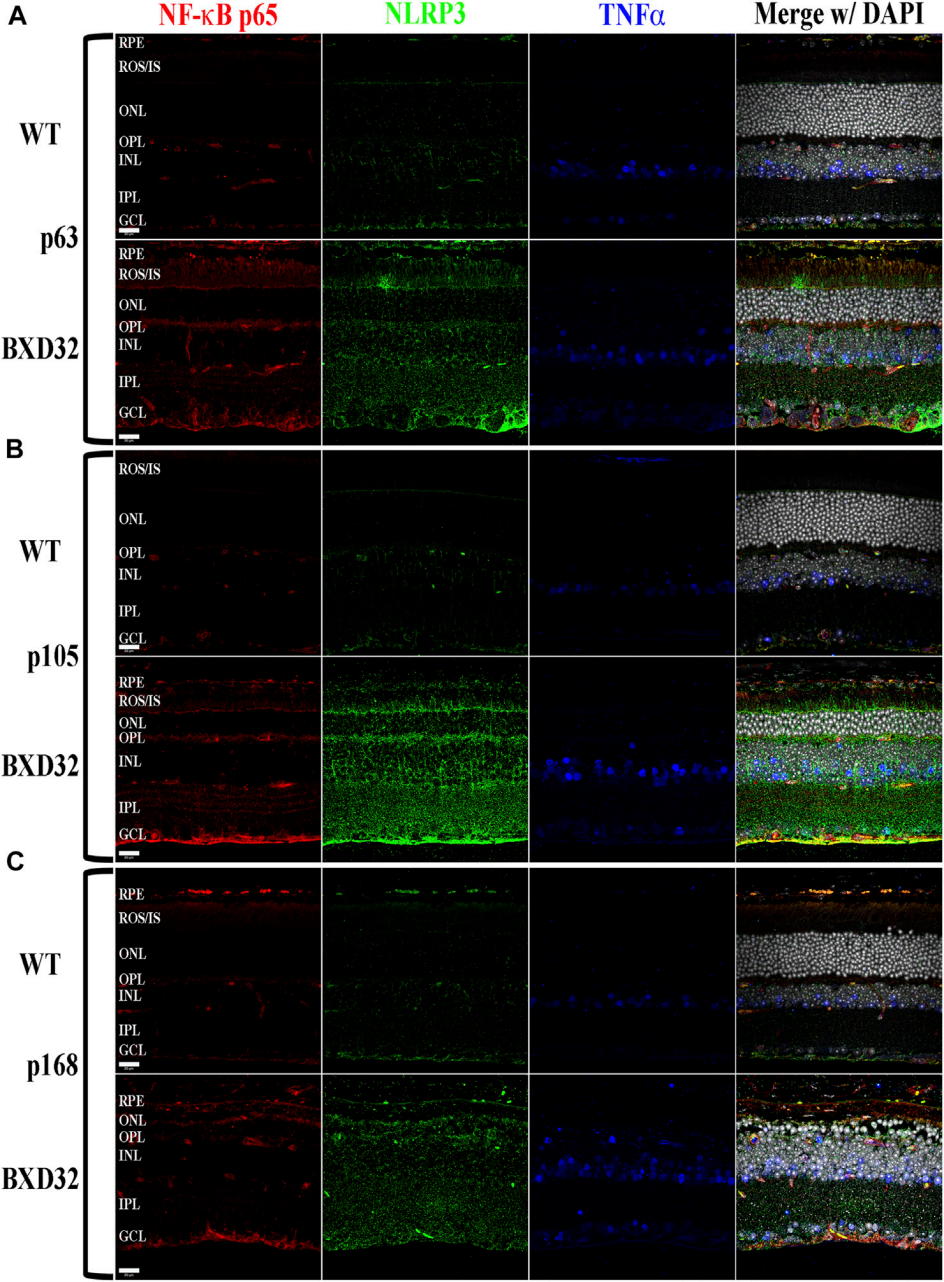
The proinflammatory cytokine TNFα and one of its cognate pathways, the NF-κB/NLRP3 inflammasome, are substantially upregulated and activated in the BXD32 mouse retina. Using immunolabeling for NF-κB p65 (red), NLRP3 (green), and TNFα (blue), the BXD32 mouse retina displays upregulated levels of all three proteins examined beginning as early as p63 **(A)** and continuing through p105 **(B)** and p168 **(C)**, indicating a chronic proinflammatory retinal environment. Nuclei stained with DAPI (white/grey). RPE, retinal pigment epithelium; ROS/IS, rod outer segments/inner segments; ONL, outer nuclear layer; OPL, outer plexiform layer; INL, inner nuclear layer; IPL, inner plexiform layer; GCL, ganglion cell layer. Scale bars = 20 μm.

TNFα is released from multiple retinal cell types under normal and diseased physiological conditions. When TNFα is upregulated, such as in proinflammatory conditions, it initiates a cascade through its cognate receptors to ultimately cause upregulation of inflammatory pathways including NF-κB and NLRP3 upregulation and activation (Hohmann et al., 1990; Meichle et al., 1990; Bauernfeind et al., 2009; Toma et al., 2010; Qiao et al., 2012; Hollingsworth et al., 2021). We observed that TNFα is upregulated in the BXD32 retina compared to WT and both NF-κB and NLRP3 are grossly upregulated indicating heavy proinflammatory signaling in the degenerating retina (Figure 7 and Supplementary Figure S5). This upregulation is high regardless of age.

**FIGURE 8:**
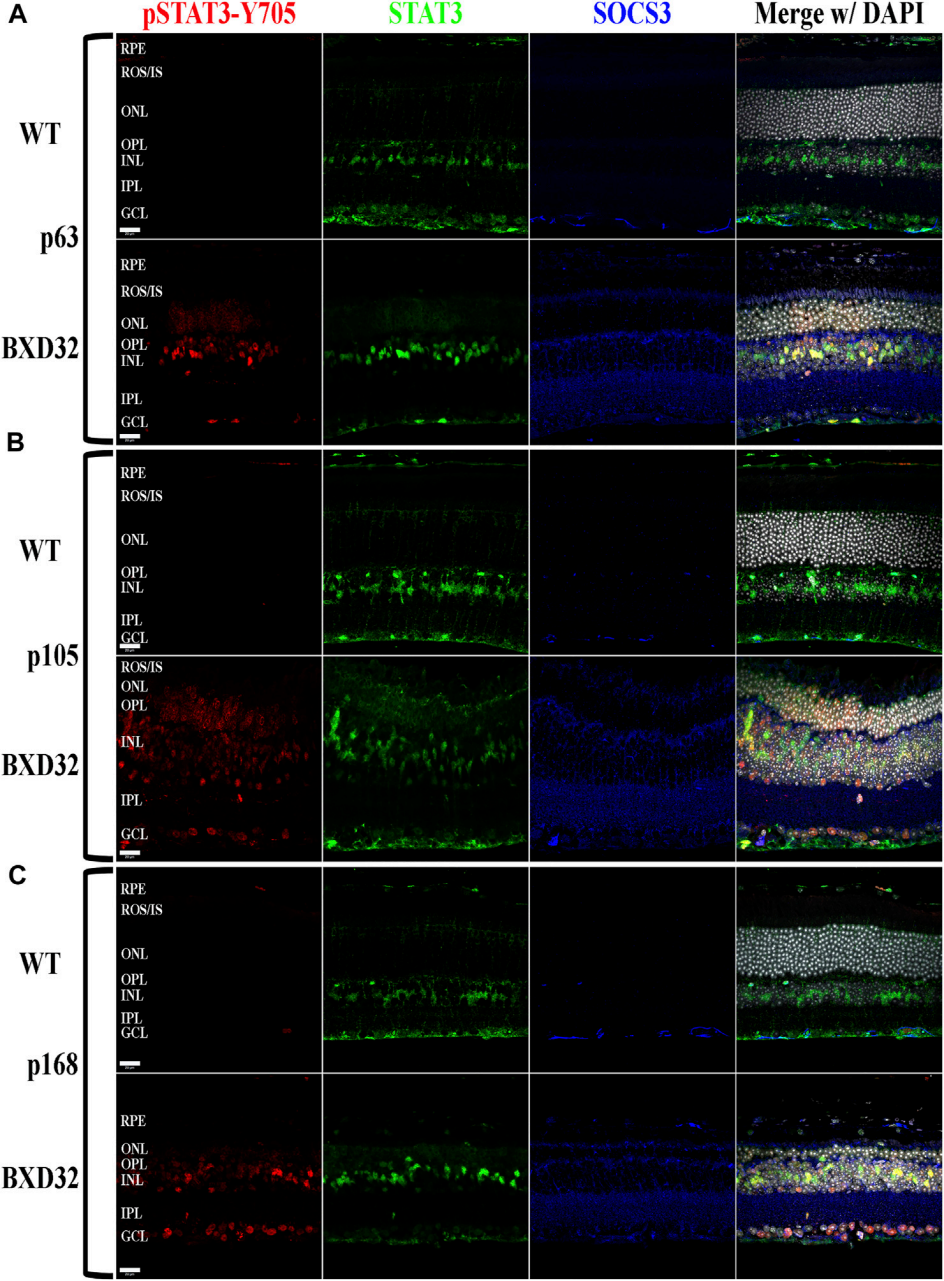
Activation of STAT3 by JAK phosphorylation is observed in the BXD32 mouse retina. Using immunolabeling for pSTAT3 at tyrosine 705 (pSTAT3-Y705, red), total STAT3 (green) and the inhibitory protein SOCS3 (blue), the BXD32 mouse retina exhibits both increased expression of total STAT3 and the inhibitory protein SOCS3, while also having substantial STAT3-Y705 phosphorylation which increases with age from p63 **(A)** to p105 **(B)** up through p168 **(C)**. This pSTAT3 labeling is observed in multiple cell layers of the retina including the ONL, INL and GCL. Nuclei stained with DAPI (white/grey). RPE, retinal pigment epithelium; ROS/IS, rod outer segments/inner segments; ONL, outer nuclear layer; OPL, outer plexiform layer; INL, inner nuclear layer; IPL, inner plexiform layer; GCL, ganglion cell layer. Scale bars = 20 μm.

**FIGURE 9:**
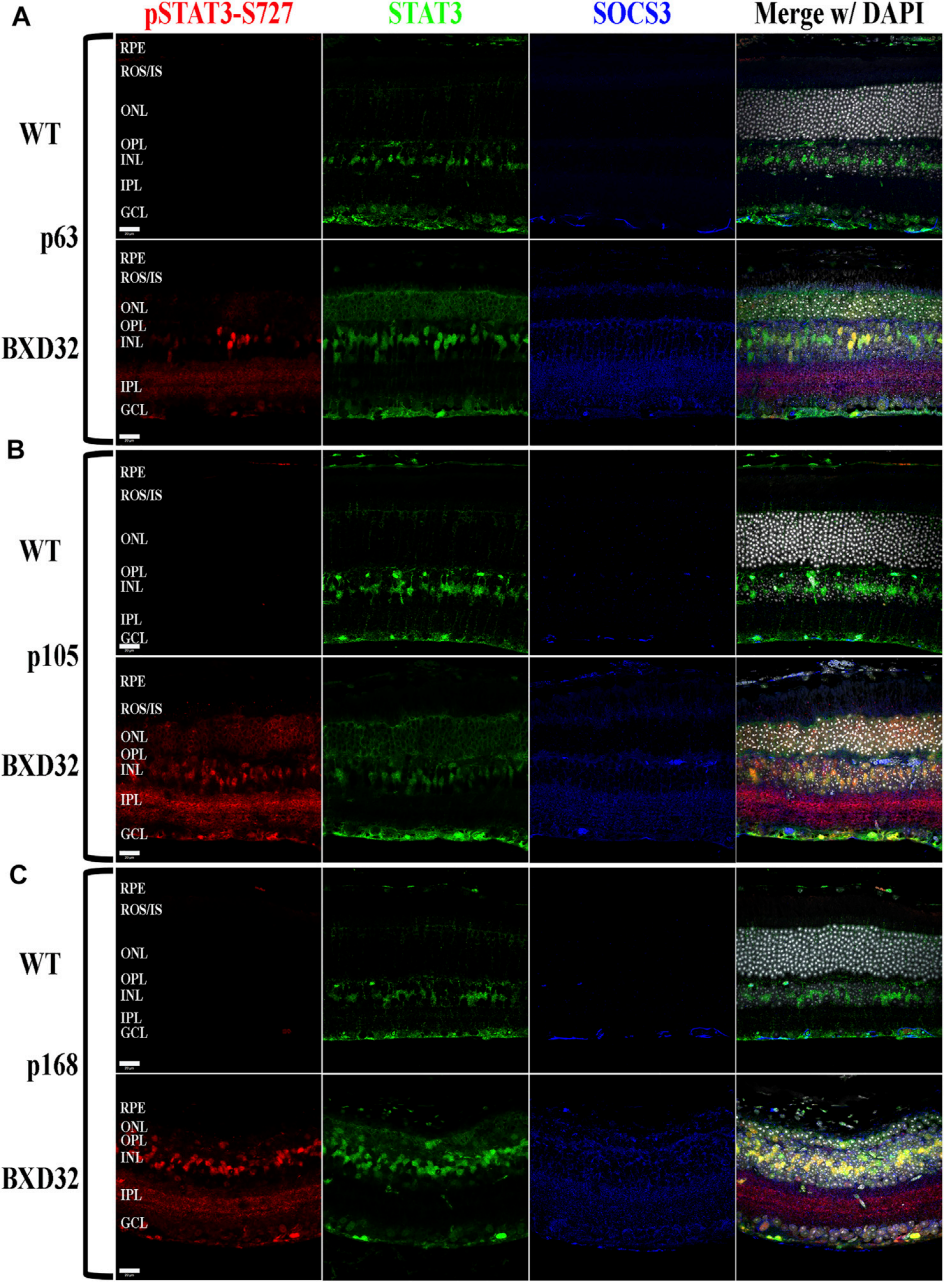
BXD32 mouse retinas also exhibit STAT3 phosphorylation at serine 727, a multifunctional phosphorylation site. Using immunolabeling for pSTAT3 at serine 727 (pSTAT3-S727, red), total STAT3 (green), and SOCS3 (blue), it is observed that BXD32 retinas have excessive phosphorylation on STAT3-S727, a phosphorylation site serving multiple functions, two of which are the initiation of dephosphorylation of Y705, the inactivation step of STAT3, and upregulation of the mRNA for SOCS3. This upregulation can be observed in p63 **(A)**, p105 **(B)**, and p168 **(C)** retinas. The upregulation of both STAT3 and SOCS3 can also be observed in these sections. Nuclei stained with DAPI (white/grey). RPE, retinal pigment epithelium; ROS/IS, rod outer segments/inner segments; ONL, outer nuclear layer; OPL, outer plexiform layer; INL, inner nuclear layer; IPL, inner plexiform layer; GCL, ganglion cell layer. Scale bars = 20 μm.

**FIGURE 10:**
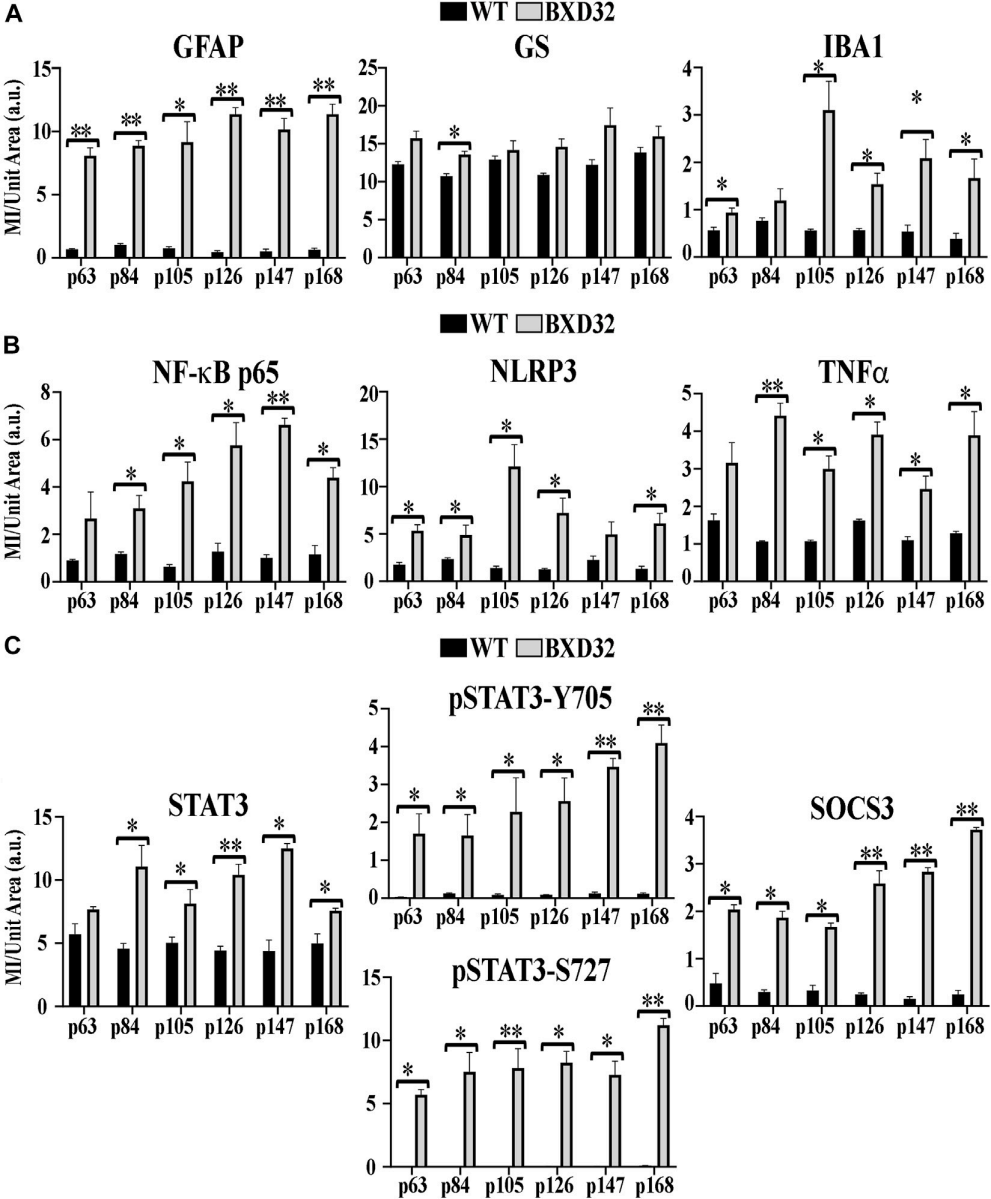
Quantification of fIHC. Using ImageJ, mean intensity (MI) per unit area in arbitrary units (a.u.) was assessed for fIHC labeling of GFAP, GS, and IBA1 **(A)**; NF-κB, NLRP3, and TNFα **(B)**; and STAT3, pSTAT3-Y705 and S727, and SOCS3 **(C)** in WT (black) and BXD32 (grey) mouse retinas with age. Error bars = ±SEM. *, *p* < 0.05; **, *p* < 0.005.

The Janus kinase/signal transducer and activator of transcription (JAK/STAT) pathway is known to be activated by proinflammatory cytokines such as interleukins 1 and 6 as well as pro-survival/cell division factors such as leukemia inhibitory factor (LIF) and, depending on the ligand, the pathway serves different functions (Nakashima and Taga, 1998). STAT3, which is phosphorylated by JAK, is capable of being phosphorylated at Tyr705 and Ser727, with the two sites serving varying functions. The Tyr705 site tends to induce activation, dimerization, and transcriptional activity, whereas the S727 site has been implicated in the deactivation of the dimerized STAT3, as well as other functions such as immunity and cell division (Lim and Cao, 1999; Murray, 2007; Wakahara et al., 2012; Balic et al., 2020; Yang et al., 2020). To examine for activation of the JAK/STAT signaling pathway, we performed fIHC for both pSTAT3-Tyr705 and pSTAT3-Ser727 along with total STAT3 and the negative regulator of the pathway, SOCS3. We observed dramatic upregulation of pSTAT3-Tyr705 with age (Figure 8 and Supplementary Figure S6) and a concurrent phosphorylation at S727 (Figure 9 and Supplementary Figure S7 **)**. As expected, the pSTAT3-Tyr705 localizes to the nuclei of the various retinal cell types while the pSTAT3-Ser727 can be found in both the nuclei as well as other non-nuclear regions such as the IPL. Interestingly, the total STAT3 and pSTAT3-Ser727 do not co-label unless present in the nucleus. As the epitope for the STAT3 antibody (peptide surrounding Gln692) is prior to the Ser727 site, the lack of co-labeling seems confounding; however, previous works have shown that STAT3 is proteolyzed by multiple caspases, enzymes which we know are involved in apoptosis and inflammation, both processes of which appear heavily active in the BXD32 mouse retina (Darnowski et al., 2006; Matthews et al., 2007). As there are three recognized isoforms of STAT3 (α, β, and γ, though a δ form has also been observed), but only one of them has the Ser727 site, the Ser727 labeling must be on STAT3α. As shown in Danowski, *et al,* six putative caspase recognition sites are present in the STAT3α protein, with an xxxD motif present from residues 720 to 723, immediately prior to the Ser727 site but after the STAT3 epitope. This is a quite plausible explanation for the large quantity of pSTAT3-Ser727 labeling observed outside of the nuclei. This is the first time we have observed such an intense and copious amount of STAT3 activation in a mouse model of RP as, in the rhodopsin mouse models, the pSTAT3-Tyr705 labeling was confined or mostly confined to the Müller cells and photoreceptors; however, as observed in the BXD32 retina, the STAT3 activation broadens to nearly every retinal cell type as early as p63 with a concomitant propagation across the entire retinal section by p147. We also observe, in contrast to what was observed in the rhodopsin-mediated RP mouse models, a substantial increase in the levels of SOCS3 in the BXD32 mouse retina compared to WT retinas, indicating, in conjunction with phosphorylation at Ser727, a possible attempt to lessen or control the proinflammatory signaling. Using ImageJ, fluorescent intensities were quantified from confocal images for all immunolabels and the results expressed as the means ± SEM (Figure 10).

## Discussion

Progressive RDs affect a myriad of diverse populations. For instance, AMD preferentially afflicts elderly white females from western cultures while RP and LCA primarily affect young and middle-aged adults and children/teenagers, respectively, from regions with more consanguineous families (den Hollander et al., 2008; Jordan et al., 2021; Rho et al., 2021). The heterogeneity of RDs has, to date, been one of the most in surmountable factors in producing therapeutics capable of tackling multiple types of RDs. The primarily genetic etiologies and age of disease onsets of RP and LCA make gene therapy for these diseases an arduous, complicated task. To exemplify this, one can examine the variety of modes of inheritance associated with RP. While autosomal recessive or X-linked recessive RP can be somewhat straightforward (i.e. replacing the mutated gene product with the correct gene product), treating the autosomal dominant or maternally inherited forms becomes significantly more challenging as supplying the correct protein product doesn’t correct the issue as one copy of the mutated gene is enough to cause the degeneration (Dryja et al., 1990; Rivolta et al., 2002; Ferrari et al., 2011; Gorbatyuk et al., 2012; Hollingsworth and Gross, 2012; Orlans et al., 2020; O'Neal and Luther, 2021). Thus, a more intricate and sophisticated therapy is necessary to reduce expression of the mutated gene, while possibly also enhancing expression of the correct gene. LCA, though always recessively inherited, presents a different problem. While gene therapy for one form LCA has already been FDA-approved (LCA caused by *RPE65* mutations), one must now consider the age of the subjects that need to be treated (Acland et al., 2001; Narfstrom et al., 2003). For LCA to be effectively countered and the highest amount of vision preserved for the patients, they would need to be treated as infants/young children or even *in utero* as LCA has a disease onset of birth and often a high rate of progression (den Hollander et al., 2008). In the case of AMD, patients may not even know they have an RD until the degeneration has already set in. By this point, the lost vision cannot be restored as the photoreceptors and/or RPE in that region have already degenerated and the disease progression cannot, as of yet, be halted or even hindered in many cases, especially in the dry form (Chintalapudi et al., 2019; Rho et al., 2021).

In describing the many pitfalls associated with RD heterogeneity, one can easily lose hope in the prospects of treating these debilitating diseases; nevertheless, RDs do, in fact, have common factors. One of these, and currently one of the most promising targets, is retinal inflammation. All RDs present with some form of inflammatory phenotype, be it association with the complement system in AMD, upregulation of cytokine release and their downstream pathways in RP and LCA, or overactive aberrantly functioning monocytes, RDs are now seemingly always associated with a proinflammatory retinal uptick (Iannaccone et al., 2015; Iannaccone et al., 2017; Hollingsworth and Gross, 2020; Hollingsworth et al., 2021; Jordan et al., 2021; Rho et al., 2021). Targeting these molecules, cells and pathways could likely allow for the ability to slow the progression of the degenerative phenotypes associated with progressive RDs. The fact that many of the proinflammatory cytokines and chemokines observed in RD retinas are also found to be associated with cancer and autoinflammatory conditions, finding already FDA-approved therapeutics targeting these molecules can be as simple as doing an Internet search such as on DrugCentral.com. For example, in the mouse models of RP referenced previously (as well as the BXD32 mouse described in this work), upregulation of the JAK/STAT pathway was observed and this fell heavily in the Müller cells (Hollingsworth and Gross, 2020; Hollingsworth et al., 2021). By searching DrugCentral.com, our group discovered the drug upadacitinib, an FDA-approved JAK inhibitor produced by Abbvie, that we tested with positive results on LIF-activated rat retinal Müller cells (rMC-1 cells) (Hollingsworth et al., 2021).

Within this manuscript, we describe a novel polygenic model of IRD, the BXD32 mouse strain. As we have shown, the retinas of these mice degenerate at varied rates in different geographic regions of the retina with regions from the central to peripheral and superior to inferior retina degenerating more rapidly with an onset as early as p63. Further preliminary work looking at a younger age has shown that apoptosis of photoreceptors is already occurring at p42 (n = 1, Supplementary Figure S8 **)**. As we continue to investigate the pathogenetic mechanisms governing the degeneration observed in these mice, younger ages will be inspected to determine the specific age of onset. As these mice represent a spontaneous IRD, they could make a more accurate model of the human condition, especially in cases of polygenic IRDs, which have been shown to occur in human patients (Bergsma and Brown, 1975; Kajiwara et al., 1994; Katsanis et al., 2001; Burkard et al., 2018), when compared to the more artificial transgenic and knock-in models of IRDs. In the BXD32 mouse retina, we documented numerous markers of proinflammatory pathway activation, of which one striking observation was macrophages performing both proper and aberrant phagocytosis of retinal cells. Some of these cells were labeled for TUNEL, a marker for apoptosis; however, several cells observed being phagocytosed at all ages lacked TUNEL labeling, indicating the process of phagoptosis, previously described in other models of RP (Brown and Neher, 2012; Brown and Neher, 2014; Li et al., 2015; Zhao et al., 2015; Hollingsworth et al., 2021). This process is likely a strong underlying cause of the rapid pace of degeneration. We have, in addition, shown upregulation of numerous proinflammatory factors and pathways. TNFα is secreted by multiple cell types including macrophages and glial cells. This allows for initiation of pathways involving activation of NF-κB and the NLRP3 inflammasome (Collart et al., 1990; Hohmann et al., 1990; Meichle et al., 1990). Interestingly, these pathways can create a positive feedback loop which ultimately causes more secretion of TNFα (Collart et al., 1990; Hohmann et al., 1990; Meichle et al., 1990; Shannon et al., 1990). We also show that the JAK/STAT signaling pathway is activated in the BXD32 mouse retina and that this activation ultimately spans the entire retina. At this time, we are uncertain as to which cytokine(s) are initiating this pathway as many can, including IL-6, LIF, and others (Levy and Lee, 2002; Murray, 2007). We have future experiments planned to pin down the cytokine(s) in order to better understand the purpose of STAT3 activation. In addition to the Tyr705 phosphorylation which activates STAT3, we also observed a dramatic increase in phosphorylation at Ser727, a site conferring multiple different properties. Ser727 is typically only phosphorylated after Tyr705 as Ser727 phosphorylation is an initiation step to deactivate STAT3 by dephosphorylating Tyr705 and inducing upregulation of the SOCS3 mRNA, the inhibitor of this pathway (Lim and Cao, 1999; Wakahara et al., 2012; Yang et al., 2020). We also found it quite interesting that the Ser727 phosphorylation labeling existed outside of the nucleus and did not colocalize with total STAT3 in these regions. As we mentioned, this could be explained by caspase activity targeting an xxxD caspase cleavage motif near the C-terminus of STAT3 (Darnowski et al., 2006; Matthews et al., 2007). Future plans for the BXD32 mouse include experiments to pin down the exact genetic etiologies resulting in the degenerative phenotype to assess its applicability as a preclinical model for testing of therapeutics.

Utilizing pre-clinical animal models of RDs such as the BXD32 mouse and the therapeutic resources already available to the scientific community, treating RDs by targeting inflammation has become a pivotal and highly explored area of basic and clinical research in ophthalmology. Thus, it is our goal, to use our pre-clinical mouse models including BXD32 to better understand the pathophysiology of these diseases and discover more efficacious treatments and ultimately, cures, for progressive RDs.

## Data Availability

The raw data supporting the conclusions of this article will be made available by the authors, without undue reservation.
