# Supplementary material for: Chronic Proinflammatory Signaling Accelerates the Rate of Degeneration in a Spontaneous Polygenic Model of Inherited Retinal Dystrophy
Source: Front Pharmacol. 2022 Mar 21;13:839424. doi: 10.3389/fphar.2022.839424 (PMC8978607; doi:10.3389/fphar.2022.839424)
Supplement: Supplementary file 1 [file DataSheet1.PDF]

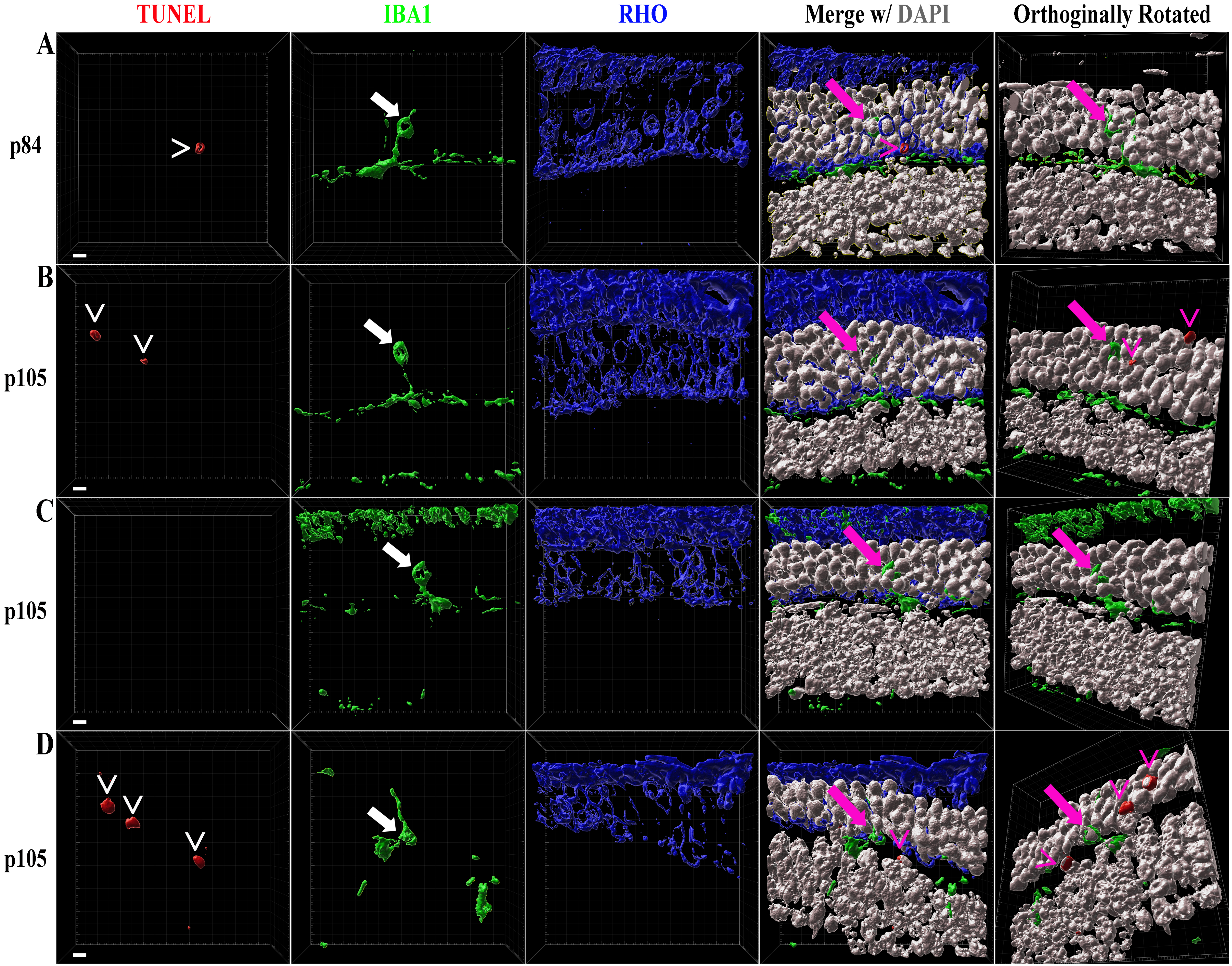

Supplementary Figure 4. The BXD32 retina exhibits phagoptosis using 3D modeling. Using TUNEL labeling (**red**) for apoptotic nuclei (arrowheads, >) and immunolabeling for monocytes (IBA1, **green**) and photoreceptors (RHO, **blue**) at p84 **(A)** and p105 **(B-D)**, followed by subsequent 3D reconstruction, presumed microglia can be observed migrating to the ONL and phagocytosing non-apoptosing photoreceptor nuclei (arrows, ➡), an abberant function of these leukocytes. Rotated images show the monocytic enveloping of photoreceptor nuclei from the reverse side of the Z-stack, conclusively demonstrating phagoptosis. Scale bars = 5 μm.
