# Supplementary figures and images for: Chronic Proinflammatory Signaling Accelerates the Rate of Degeneration in a Spontaneous Polygenic Model of Inherited Retinal Dystrophy
Source: Front Pharmacol. 2022 Mar 21;13:839424. doi: 10.3389/fphar.2022.839424 (PMC8978607; doi:10.3389/fphar.2022.839424)

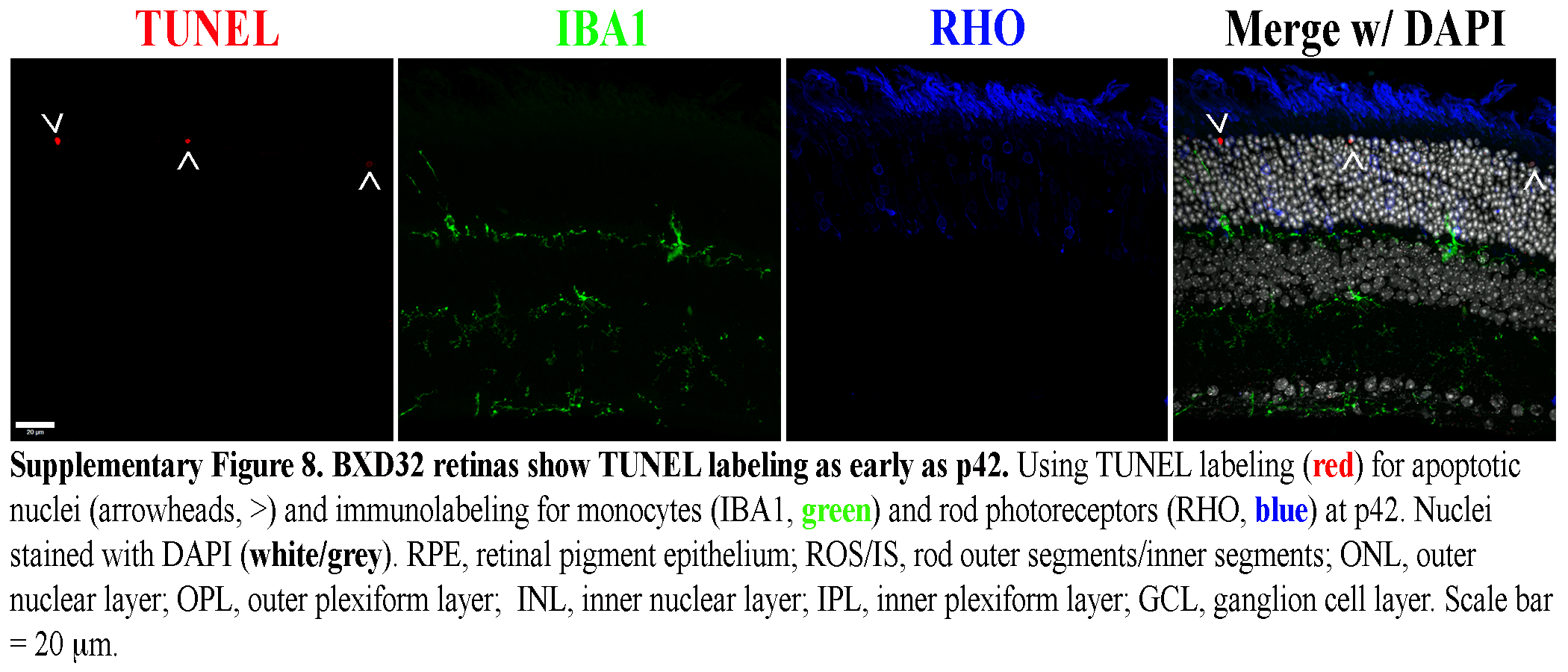

Supplement: Supplementary file 2 [file DataSheet2.zip › Supplementary Figures/Supplementary Figure 8.tif]

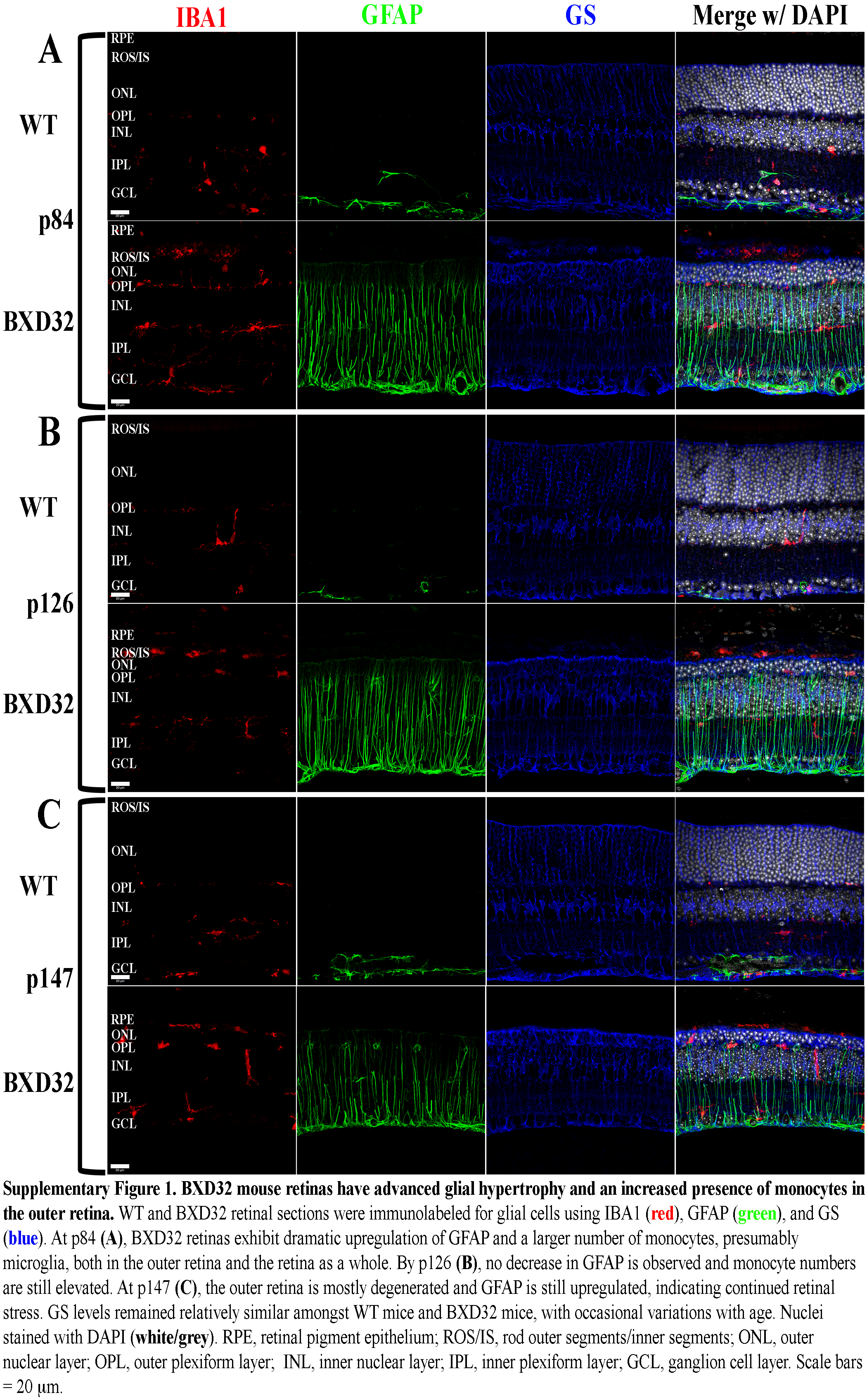

Supplement: Supplementary file 2 [file DataSheet2.zip › Supplementary Figures/Supplementary Figure 1.tif]

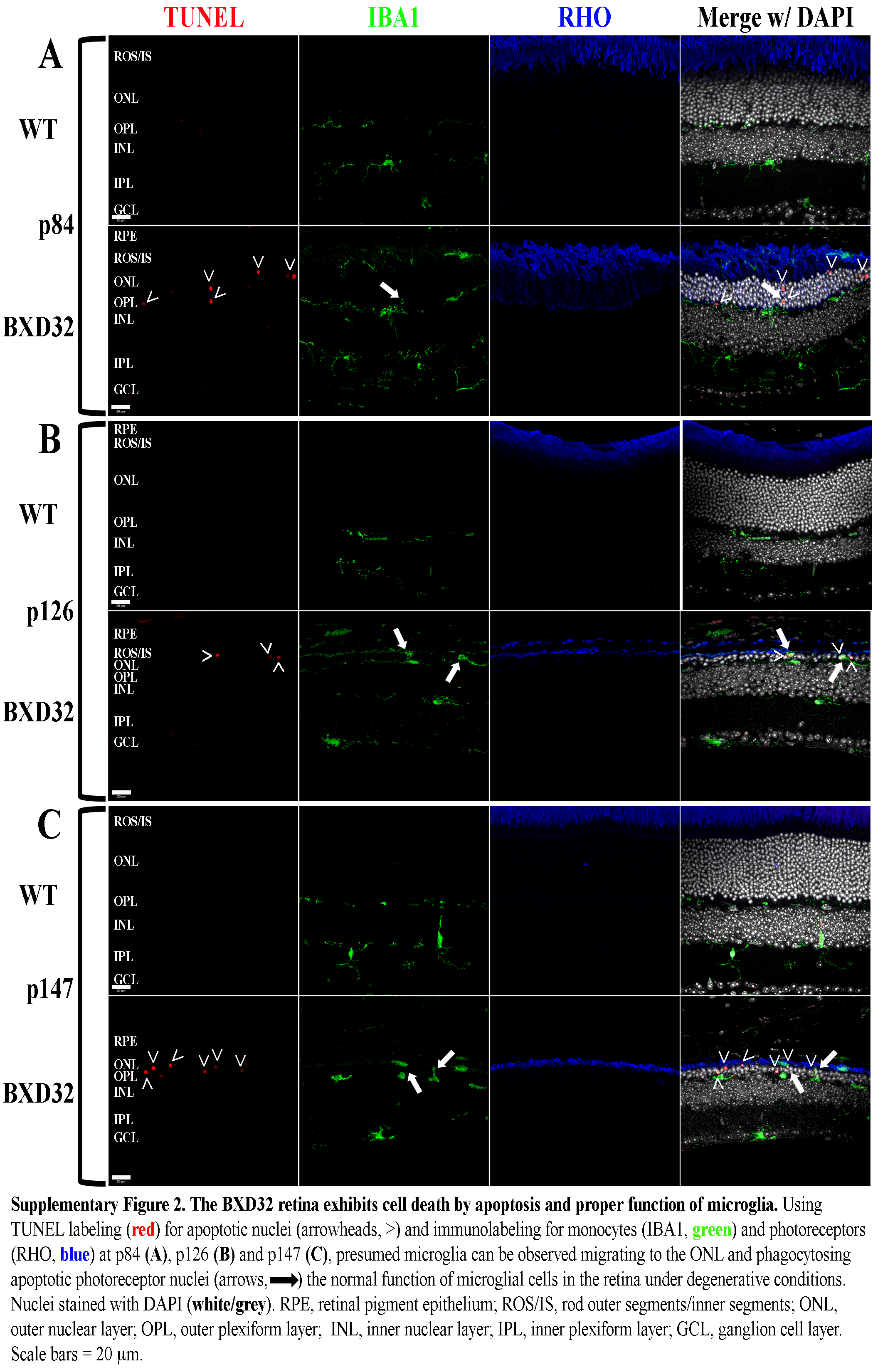

Supplement: Supplementary file 2 [file DataSheet2.zip › Supplementary Figures/Supplementary Figure 2.tif]

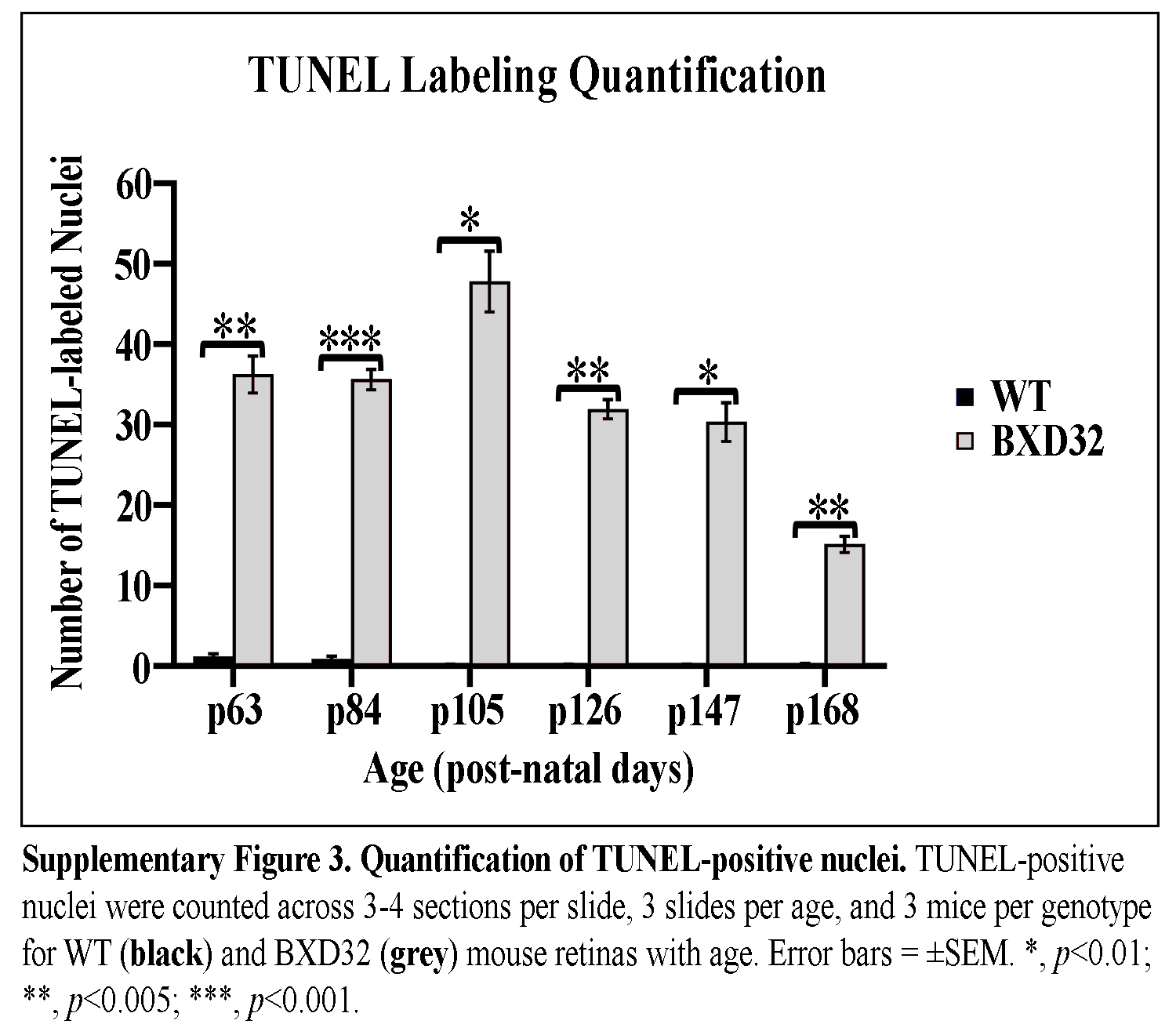

Supplement: Supplementary file 2 [file DataSheet2.zip › Supplementary Figures/Supplementary Figure 3.tif]

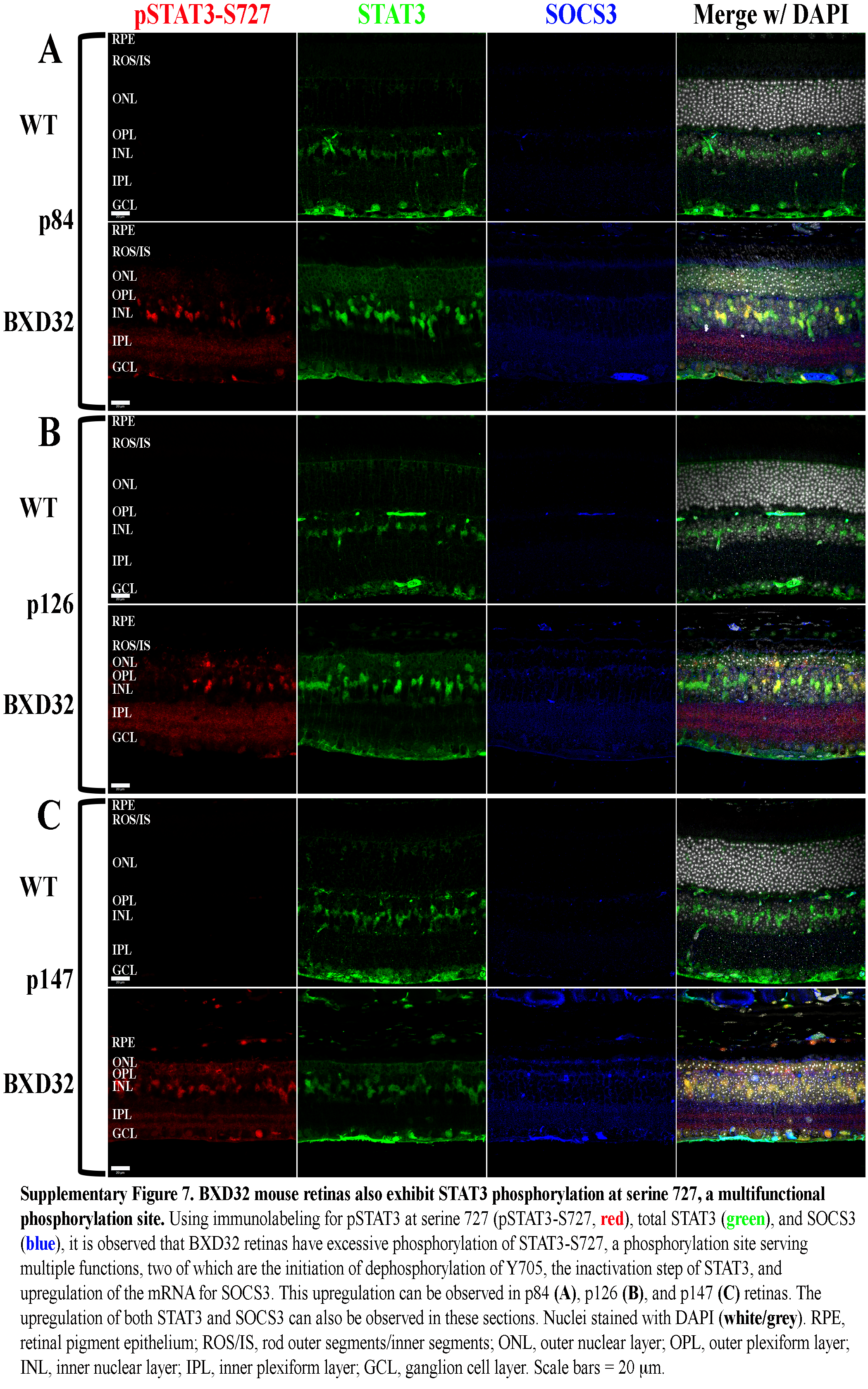

Supplement: Supplementary file 2 [file DataSheet2.zip › Supplementary Figures/Supplementary Figure 7.tif]

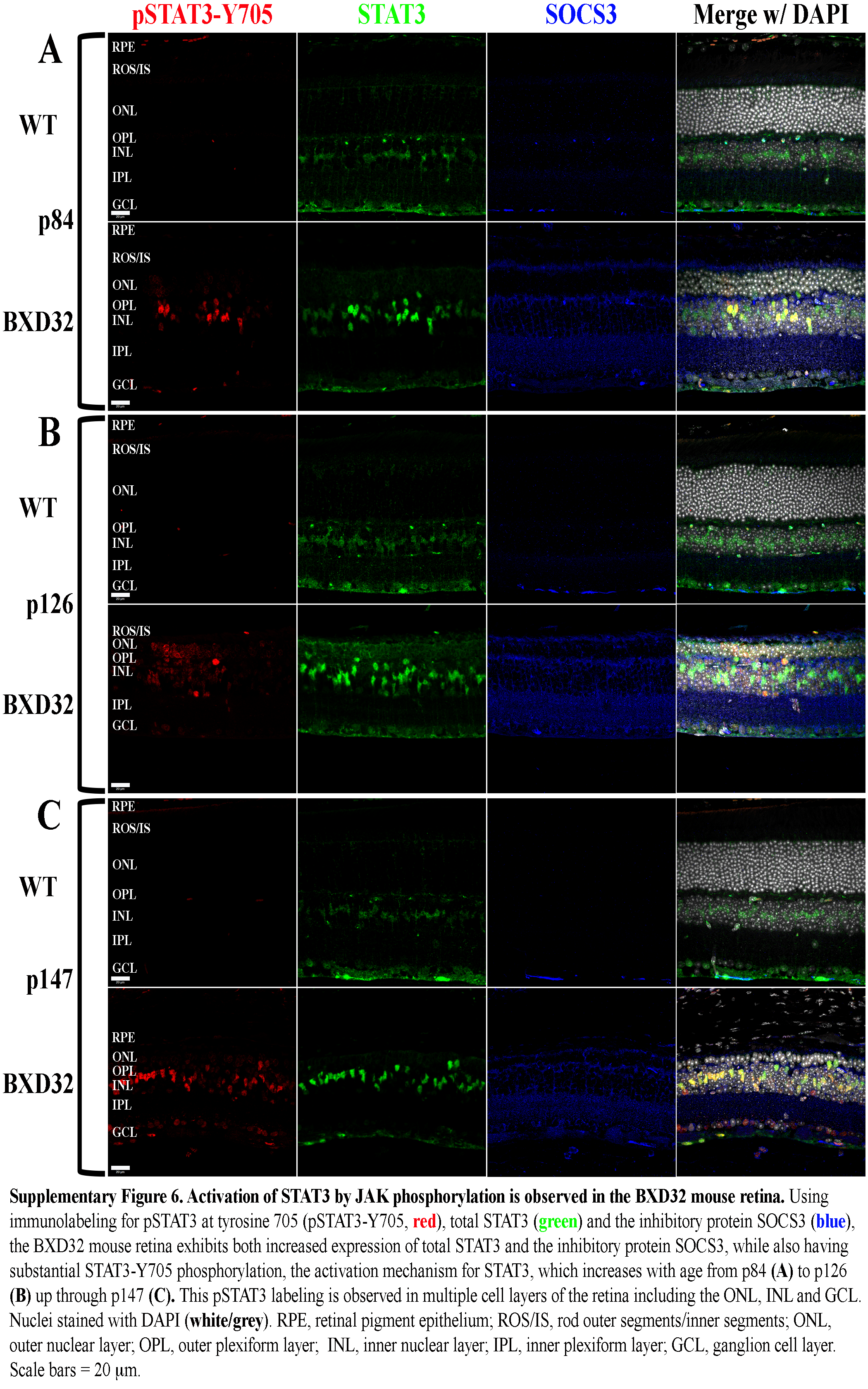

Supplement: Supplementary file 2 [file DataSheet2.zip › Supplementary Figures/Supplementary Figure 6.tif]

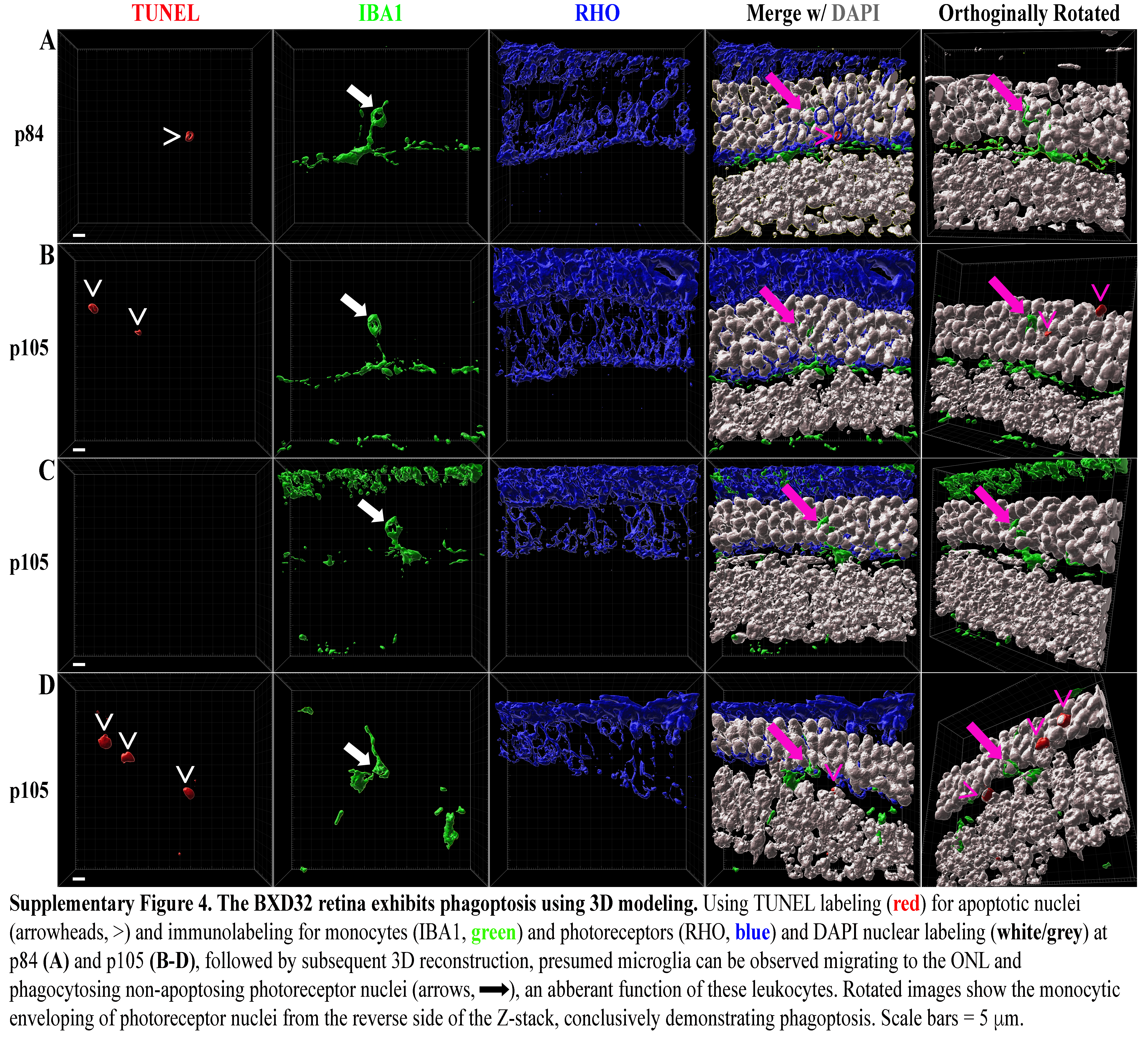

Supplement: Supplementary file 2 [file DataSheet2.zip › Supplementary Figures/Supplementary Figure 4.tif]

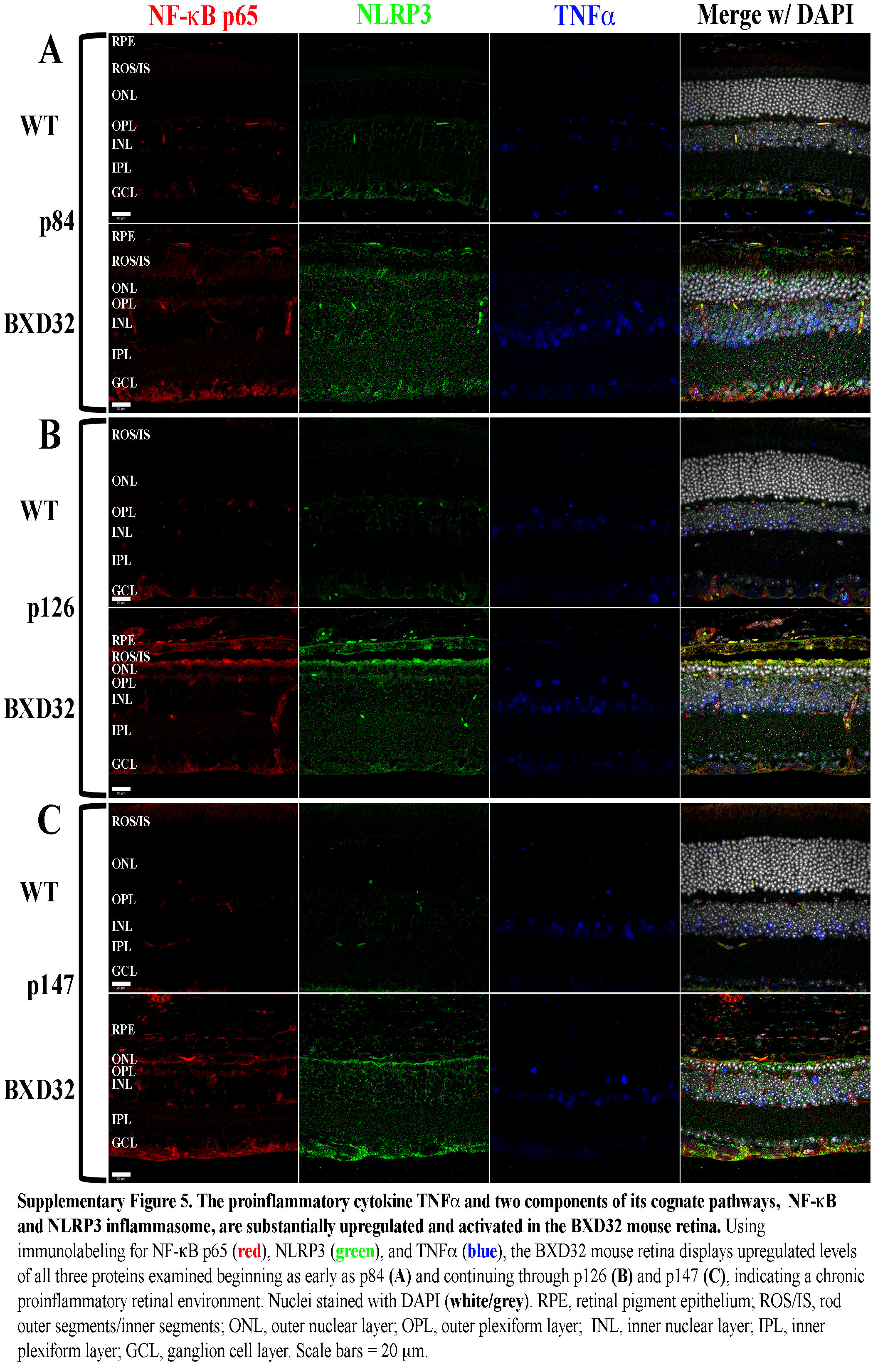

Supplement: Supplementary file 2 [file DataSheet2.zip › Supplementary Figures/Supplementary Figure 5.tif]
